# Upper Limb-Salvage Surgery in Pediatric Patients with Malignant Bone Tumors Using Microsurgical Free Flaps: Long-Term Follow-Up

**DOI:** 10.3390/biomedicines13071638

**Published:** 2025-07-04

**Authors:** Jakub Opyrchał, Bartosz Pachuta, Daniel Bula, Krzysztof Dowgierd, Dominika Krakowczyk, Anna Raciborska, Łukasz Krakowczyk

**Affiliations:** 1 2nd Department of Oncologic Surgery, Maria Sklodowska Curie Memorial National Cancer Center, 44-100 Gliwice, Poland; 2 Department of Oncology and Surgical Oncology for Children and Youth, Institute of Mother and Child, 01-211 Warsaw, Poland; 3 Department of Clinical Pediatrics, Head and Neck Surgery Clinic for Children and Young Adults, University of Warmia and Mazury, 10-709 Olsztyn, Poland; krzysztofdowgierd@gmail.com; 4 Pediatric Surgery and Urological Department, Upper Silesian Child Health Center in Katowice, Silesian University of Medicine, 40-052 Katowice, Poland

**Keywords:** osteosarcoma, limb-salvage surgery, forearm, reconstructive surgery, free flap

## Abstract

**Background**: Primary malignant bone tumors among adolescent patients are most commonly associated with burdensome surgeries that can severely affect young patients’ early life. To this day, despite available autologous tissue donor sites, cement spacers or endoprostheses are still most commonly used as a form of reconstruction of post-resection defects. **Methods**: The study group includes 20 adolescent patients diagnosed with Osteosarcoma or Ewing Sarcoma involving the upper limbs. The inclusion criteria were as follows: primary malignant bone tumors sensitive to neoadjuvant chemotherapy, tumors not infiltrating major blood vessels and nerves, and the appliance of the microsurgical free flap as a reconstructive method. Poor tumor response to neodajuvant chemotherapy or patients with incomplete follow-up were excluded from this study. To achieve the functional reconstruction of post-resection defects, fibula free flaps were applied. In cases of resection, including the metaphysis of a long bone, a modification of the flap harvest was applied in order to prevent arthrodesis. The MSTS (Musculoskeletal Tumor Society Scoring System) scale was used as a functional outcome measurement tool. **Results**: The reported outcomes of this study prove the efficiency of the treatment’s approach of combining the resection of the tumor with subsequent microsurgical restoration with the use of autologous tissues. The average score on the MSTS scale, which assesses the functional outcome, was 26.8/30 points, which indicates great motor outcomes. There were no reports of local recurrence during follow-up. **Conclusions**: Patients with primary malignant bone tumors in the upper limbs can benefit from microsurgical techniques, which are highly customized; effective; and give sufficient functionality following extensive resection.

## 1. Introduction

**Figure 1:**
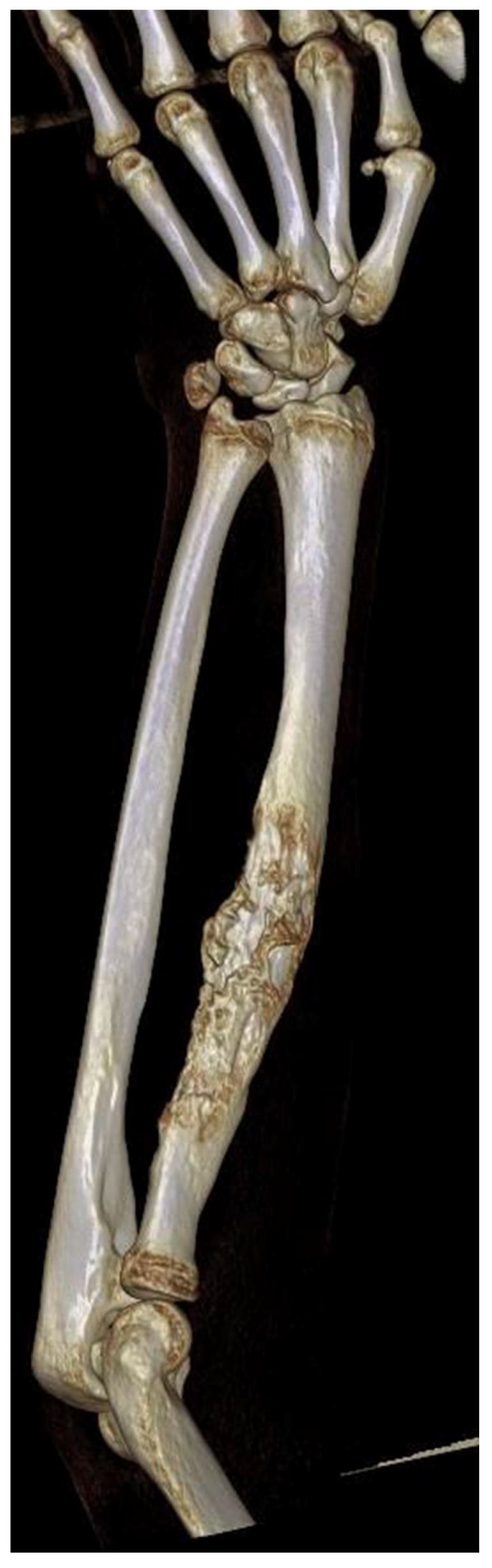
Three-dimensional reconstruction of a Computed Tomography (CT) scan of a patient diagnosed with Ewing Sarcoma of the left radius.

Within the last few decades, the field of musculoskeletal oncology has made vast strides in terms of the curability of patients diagnosed with primary malignant bone tumors and the possibility of preserving proper functionality of the limb. From an era where amputations were the main treatment option, we have proceeded to a period where limb salvage is as significant as the oncological outcome, especially when it comes to younger patients, for whom full motor reconvalescence is of paramount importance. These days, almost three-quarters of patients diagnosed with localized bone tumors are subject to limb-salvage procedures (Figure 1). Nevertheless, some researchers believe that amputation can help in mitigating the risk of complications and prevent growth-related problems [1,2]. Regarding the primary malignant bone tumors, Osteosarcoma, Chondrosarcoma, and Ewing Sarcoma are the most common histopathological diagnoses in the group of adolescent patients [3].

Currently, modern surgical techniques meet the above criteria and, in adequately qualified patients, allow for reconstruction of the post-resection defects without the use of artificial materials, relying on the patient’s own tissues (vascularized autografts). Due to the possibilities offered by microsurgery, we are able to reconstruct both bone defects as well as soft tissue deficiency with transferred vascularized tissues, harvested from a distant donor site, without carrying any additional long-term burden for the treated limb [4,5].

Throughout the years, there was a huge hope placed in massive bone allografts as an “intermediate type” of reconstruction, classified somewhere between biological reconstructions and those involving the use of artificial materials. As many studies showed, when the massive allografts were used as an independent reconstructive method, only a small percentage of the bone transferred becomes revascularised, while most parts frequently remain necrotic and act like a “biologic spacer”. There is also a long list of complications related to the appliance of massive bone allografts, such as infections, nonunions, and postoperative fractures [6,7,8].

In contrast, the vascularized bone grafts, in particular, the fibula free flap (FFF) as a preferred flap for larger bone defects (>8cm), heal and incorporate with the host bone remarkably differently [9,10]. Well-perfused bone grafts rebuild the mechanical resistance of the limb as it creates a bone union with the stumps of the resected bone while maintaining its structure during the whole healing process thanks to its independent blood supply provided by microanastomoses [11,12,13].

Without a doubt, what should be emphasized already in the Section 1, in cases where the tumor involves the metaphysis and periarticular areas, endoprostheses are often an unavoidable solution and provide a very good functional outcome, frequently preventing joint immobilization (arthrodesis) [14,15]. Nevertheless, as it will be presented in the following sections, in some cases, even in such complicated areas, it is possible to avoid arthrodesis and reduce the amount of artificial material implemented and mitigate the risk of complications specific to endoprostheses at the same time.

Moreover, the previously raised problems specific to pediatric patients related to the technical possibility of performing the microsurgical procedures on smaller vessels are no longer problematic. Because of the development of supermicrosurgery and with advances in microsurgical materials such as smaller sutures and needles, free flap transfers within pediatric patients are no longer as challenging as they once were. Complex tissue reconstruction with the appliance of microsurgery may also expand the group of patients diagnosed with advanced sarcomas, previously considered non-operable, because it can allow a surgeon to resect more tissue (in order to achieve radical resection) without worrying about wound closure or functional results. What is more, these methods ensure wound closure with well-vascularized tissues and the use of a distant donor site that does not alter the function of an already compromised limb.

The aspect that distinguishes this study from others is the fact that most reports on upper limb reconstruction analyze patients with varying age groups, types of reconstruction, anatomic locations, diagnoses, or need for surgery, whereas this study has limited the inclusion criteria to adolescents diagnosed with primary malignant bone tumors, and in all cases, a microvascular fibula free flap with possible modification within flap harvest was used as a part of surgical management.

The design of this study is to shed new light on the results of modern limb salvage treatment of pediatric patients and the associated challenges. The aim of this analysis is to demonstrate the utility and usability of the FFF in combination with bone allografts in the pediatric reconstructive procedures of the upper extremities after limb-sparing sarcoma resections and report on the incidence of complications as well as functional outcomes.

## 2. Materials and Methods

This study reports an analysis of 20 pediatric patients who underwent oncologic resections of primary bone tumors within the upper limb and reconstruction of the defect by applying microsurgical procedures i.e., fibula free flap with possible modification within flap harvest.

Patient data collected included demographics, age, gender, histology of the tumor, adjuvant oncologic treatment, type of bone involved, length of the defect, surgical margin, and type of flap used for reconstruction, as well as the presence of local recurrence or metastases during the follow-up.

Patients were invited for check-up visits at 3-month intervals after the procedure. Check-up consisted of chest X-ray and X-ray of the treated extremity for exclusion of any asymptomatic disturbances within bone union.

As an outcome measurement, each case was scored using the Musculoskeletal Tumor Society Rating Scale (MSTS) [16,17]. The upper extremity module was composed of six items, including, among others, function and psychological status, as well as upper limb-specific characteristics such as dexterity, hand positioning, and lifting ability (muscle power). Each assessed feature was scored on a scale from 0 to 5 points, with higher results reporting better functionality. There was a total of 30 points throughout all 5 items assessed. This questionnaire was completed approximately 18 months after reconstructive surgery.

All cases were additionally analyzed in terms of complications during the early postoperative period, including infection, microvascular complications, wound dehiscences, and seroma or hematoma formation.

Moreover, during check-up visits, patients underwent imaging studies such as CT scans to rule out any recurrence of disease or metastasis for the first few years after the surgical procedure.

The above-mentioned pattern of examining patients, as well as the tests and factors taken into consideration, are drawn from another study conducted by our team on limb-sparing surgery within the lower extremities, which proved the validity of this methodology [18].

## 3. Results

### 3.1. Demographics and Pathology

A total of 20 pediatric patients underwent resection of sarcoma within the bone of the upper limb and a microsurgical reconstruction with the appliance of the fibula free flap. The most common histopathology of the tumor was Osteosarcoma in 12 patients (60%), followed by Ewing Sarcoma in 8 patients (40%). The tumor involved the radius in 13 cases (65%) and ulna in 7 cases (35%). Group characteristics are presented in Table 1. The mean age of our patients was 15.1 years (range 13–17 years). There were 9 females (45%) and 11 males (55%).

### 3.2. Treatment

The mean length of the bone reconstruction was 15.1 cm (range 11–19 cm). All patients in our study (100%) had surgical margins classified as R0 (microscopically radical excision).

**Figure 2:**
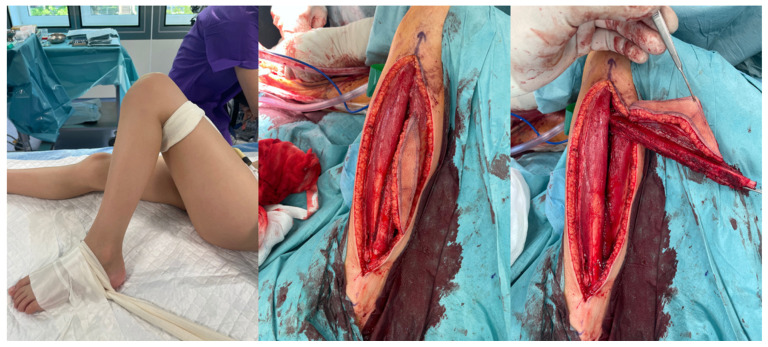
Images of fibula free flap harvest.

**Figure 3:**
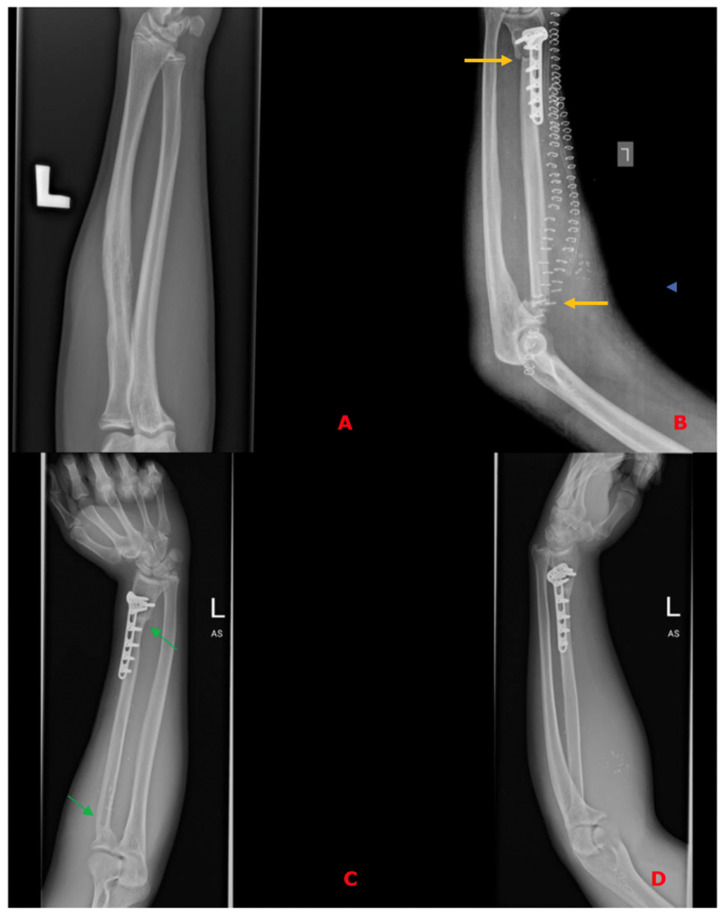
(**A**)—Preoperative X-ray of histopathologically confirmed Ewing Sarcoma of the left radius without metaphysis involvement; (**B**)—X-ray on the 1st postoperative day after resection of a specimen and reconstruction of the defect using a free fibula flap, confirming the proper axis of the reconstructed bone, bone contact points (yellow arrows), and correct positioning of the free flap. Due to the lack of technical possibility of stabilizing the proximal part using a titanium plate, stabilization with non-dissolvable sutures was used by drilling holes within the proximal part of the fibula, with subsequent thread insertion through the holes created and suture fixation through the cortical layer of the remaining proximal stump of the radius; (**C**,**D**)—Follow-up X-rays in the 14th postoperative month with visible bone union—osteointegration of the flap (green arrows) and the restored correct axis of the upper limb.

**Figure 4:**
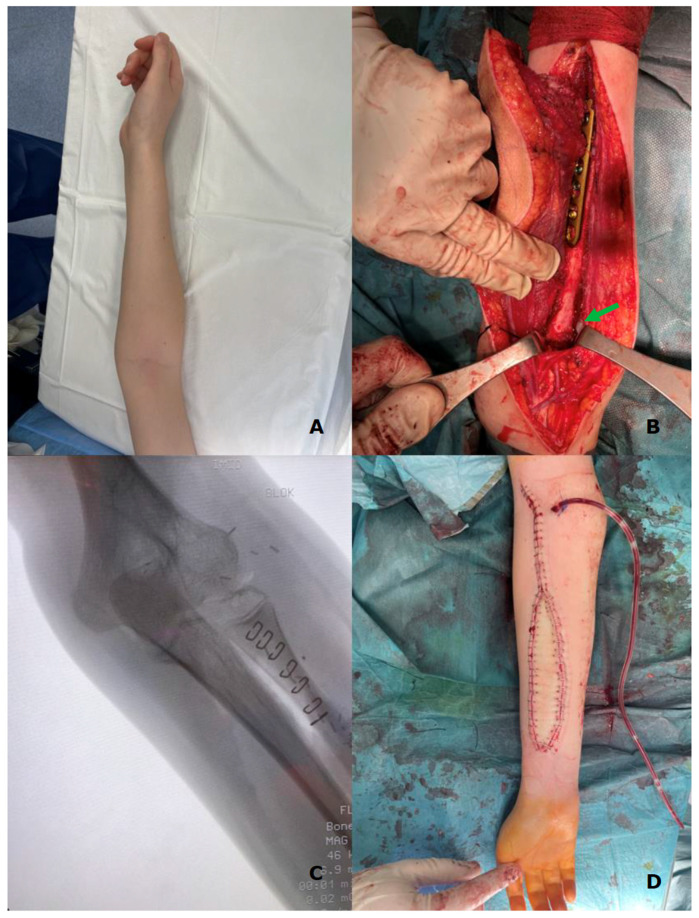
(**A**)—preoperative X-ray of the left upper limb with diagnosed Ewing Sarcoma of the radius; (**B**)—fibula free flap harvested with the fibular head (green arrow) placed within the wound bed after resection of the radial bone; (**C**)—intraoperative X-ray presenting the proper position of the fibular head within the annular ligament; and (**D**)—postoperative X-ray of the upper limb with a visible skin island of the fibula free flap (as a monitor of the appropriate perfusion of the buried part of the flap).

In all of the patients included, a fibula free flap (FFF) was applied to carry out the reconstruction with possible modifications regarding the harvest of the flap when the metaphysis was involved. The intraoperative photographs of the FFF harvest are presented in Figure 2. Most commonly, a FFF was applied to reconstruct the diaphyseal part of the long bone. In cases of the short stump of the long bone after resection part, to prevent arthrodesis, due to the lack of technical possibility of stabilizing the proximal part of the radius using a titanium plate, stabilization with non-dissolvable sutures was used by drilling holes within the proximal part of the fibula, with subsequent thread insertion through the holes created and suture fixation through the cortical layer of the remaining proximal stump of the radius (Figure 3). When the resection was not limited only to the shaft of the long bone but also included partially the proximal metaphysis of the radius, the FFF was harvested with the head of the fibula in a typical manner. During the harvest of the fibular head, the deep peroneal nerve was carefully dissected off the bone and spared, which is the crucial point for mitigating donor-site morbidity. The fibular head replaced the function of the head of the radius at the humero-radial joint and was inserted directly into the spared annular ligament of the radius, maintaining the functionality/mobility of the limb and avoiding the endoprosthesis of the humeroradial joint at the same time (Figure 4). This solution was possible due to the sparing of the annular ligament, which ensured adequate stabilization of the fibular head.

All flaps were harvested, including a skin paddle, as a monitor of transferred tissues’ viability and to avoid the necessity for performing angiographic studies to assess the blood perfusion within the fibula (Figure 2).

**Figure 5:**
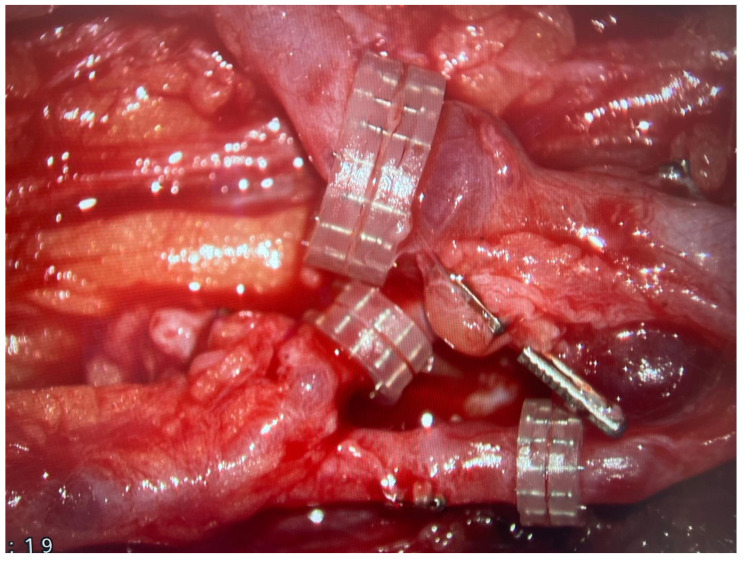
Three microvascular anastomoses performed with the use of coupler system are presented with the use of surgical microscope.

In the vast majority, the microanastomoses were performed between the FFF pedicle and branches of radial vessels. In every case, there was one arterial anastomosis and two veins connected. Veins from the FFF pedicle were anastomosed to both the superficial and deep veins of the upper limb (most commonly the cephalic or basilic vein). All venous microanastomoses were carried out using the microvascular coupler (Synovis Micro Companies Alliance, Birmingham, AL, USA). The average size of the coupler used was 3.0 mm (range 2–4 mm). (Figure 5).

**Figure 6:**
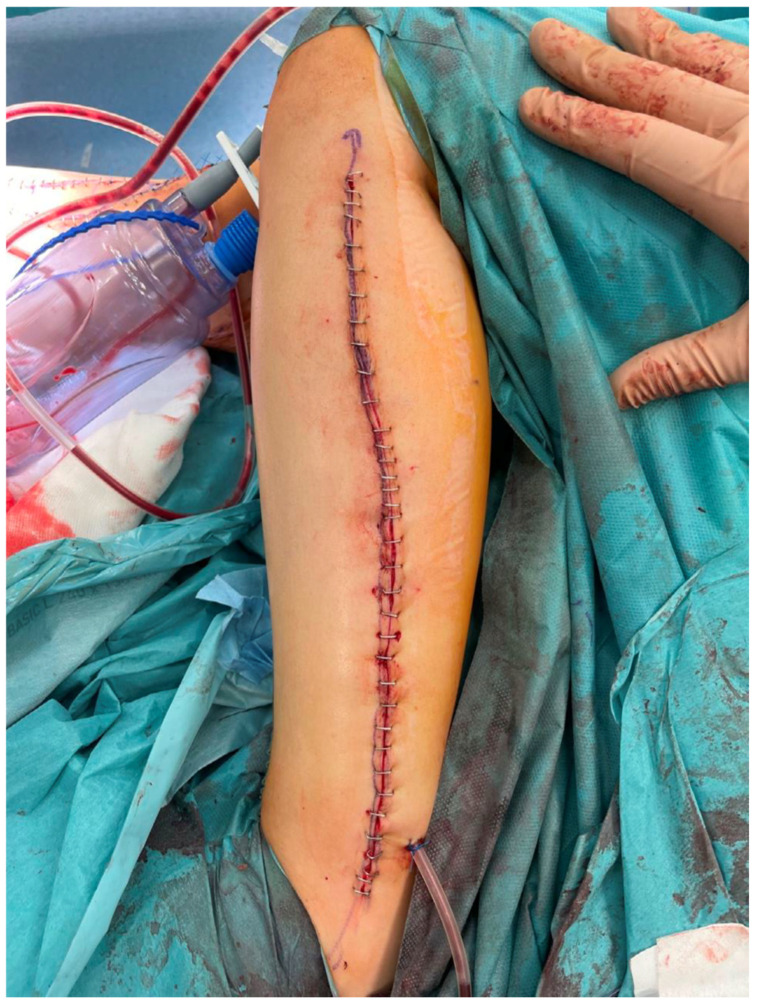
An intraoperative photograph of the donor site after harvesting a free fibula flap—the donor site was closed tension-free primarily due to the small width of the harvested skin island.

The lower legs, from which the fibula free flaps were harvested, were closed primarily without significant donor site-related complications (Figure 6).

#### 3.2.1. Flap Survival and Surgical Site Occurrences

Among the adolescent patients included in this manuscript, there was one major early postoperative complication (5%) that was not strictly related to the reconstructive part of the procedure and resulted from the need to obtain a radical excision margin—paralysis of the posterior interosseus nerve, which manifested mainly by a wrist drop. Also, two minor early postoperative complications (10%) occurred as a wound dehiscence on the eighth postoperative day, which was treated with a simple surgical resuturing. In the second case, there was venous congestion just within the skin paddle of the free flap, which was a temporary impairment that disappeared within 12 h due to upper limb elevation (reduction of forearm swelling) as well as flap heating. This data is also summarized in Table 1.

#### 3.2.2. Follow-Up (Functional Outcomes and Bone Union)

The follow-up period ranged from 27 to 45 months, with an average of 36.1 months. During the oncological observation, the check-ups revealed the presence of dissemination of the cancer to the respiratory system in two (10%) patients.

In this study group, every limb (100%) was spared. Plain X-rays were taken of the patients every two to three months. Every microvascular fibula free flap has formed a bone union at both ends, according to postoperative imaging investigations.

**Figure 7:**
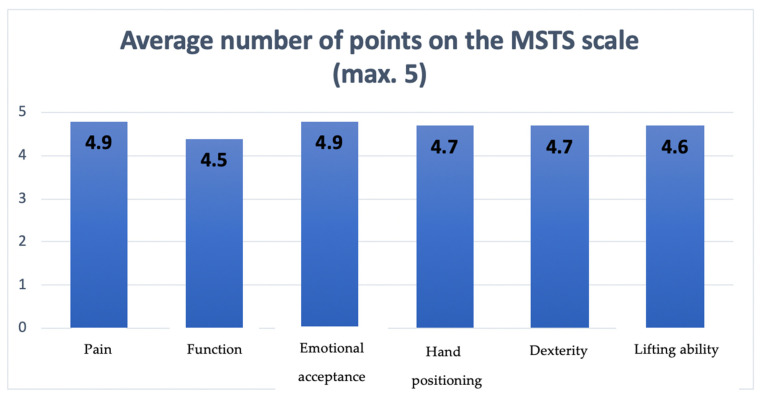
The diagram presenting points scored by patients on the MSTS scale regarding each assessed feature.

Using the functional MSTS scale, the surgical team assessed the study participants. With a range of 19–30 points, the average MSTS score was 26.8 points. Figure 7 shows the average number of points for each evaluated characteristic on the MSTS scale.

Regarding the protocol for upper limb loading, in general, patients were allowed to lift up to 2 and 4 kg, respectively, 2 and 4 months after surgery on the treated limb. After 6 months of surgery, patients were no longer restricted in lifting, with the exception of intensive strength sports. Each patient in this group achieved the intended limb loading goal.

## 4. Discussion

Primary malignant bone tumors among adolescent patients are most commonly associated with burdensome surgeries that can severely affect young patients’ early lives. To this day, despite available autologous tissue donor sites, cement spacers or endoprostheses are still most commonly used as a form of reconstruction of post-resection defects. Microsurgery is gaining considerable interest as an effective approach to functional reconstruction, limiting the amount of artificial materials used and mitigating the risk of major postoperative complications related to them at the same time.

The aim of this study is to present the results of limb-sparing treatment of advanced upper limb bone tumors in adolescent patients, using a fibular free flap, and to show that even in the case of metaphysis involvement, a satisfying functional outcome can be achieved. The obtained results present that the fibula free flap with eventual modifications can serve as a biological reconstruction with minimized risk of complications and, in some cases where it seems unavoidable, prevent arthrodesis. These results should be treated very cautiously because they refer to a relatively small group of patients. However, the modifications to the surgical technique as well as the postoperative care that have been introduced may help other centers achieve better postoperative functional outcomes, which are of paramount importance in younger patients during the period of the most dynamic motor development.

In the case of bone sarcomas, reconstructions within the limb can be carried out with the application of many different surgical approaches. Some of them are gradually falling out of clinical use due to numerous limitations and a low acceptance rate by the patient himself as well as his family. This paradigm shift in the field of reconstructive surgery happened because artificial materials, even though less morbid than biologic constructs, are reported to have a higher rate of infections and failure of mechanisms.

Nevertheless, as microsurgical procedures are characterized by quite significant difficulty, the frequency of postoperative complications in the literature reaches 38–80% [19,20,21,22].

The biggest risks that those procedures carry with them are, for sure, lack of bone union as well as possible postoperative fractures. This is due to the loads to which the reconstructed areas are subjected and the resulting need for appropriate mechanical resistance and perfusion of the tissues that are meant to produce bone union in the future. Postoperative pseudoarthrosis or fractures are complications of paramount importance because they can remarkably limit the patient’s activity and impair the rehabilitation period until bone union is achieved.

Wound healing disorders, as well as impaired flap perfusion, are the most commonly reported complications in the early postoperative period [23]. After completion of wound healing, the postoperative fractures (34%) and bone nonunion (11%) are common in the late follow-up [21]. In the report by Ruiz-Moya et al. on a group of almost 30 patients who underwent these procedures, as many as half of them required a second surgical intervention [21].

In contrast, when the endoprostheses were applied, complications specific to artificial materials implemented could be found, such as periprosthetic infections, loosening of the implant, or malfunction of the mechanical elements. Savidou et al. and Schinhan et al. also reported complications related to “soft tissues” (such as limited mobility within the extremity) as the most common in this group of patients, with rates of almost 50% [24,25].

In a study by Savidou et al. on a group of over 600 patients with endoprostheses, as many as 18.5% of them reported periprosthetic infection, and 16% of them—endoprosthetic loosening. Both complications most frequently resulted in the urgent surgical revision and removal of the endoprosthesis [24].

All these factors considered above translate into an objective assessment of the long-term effect of such treatment. In this study, we decided to use the most widely validated questionnaire in oncological orthopedics—the MSTS scale. Unfortunately, other studies available in the literature relating to limb-sparing treatment do not often use an objective method to assess long-term outcomes. Compared to a representative study conducted by Tunn et al. on 87 patients who underwent limb-sparing treatment with endoprostheses, the average MSTS score at an average of 6.5 years postoperatively was 23.1 points [26]. Our results in the form of patients obtaining 26.8/30 possible points are another confirmation of the proper quality of the presented method.

We are aware that main limitation of this study is the limited study group, which affects the rate of reported complications. Nevertheless, we believe that both technical modifications as well as the experience of the microsurgical team have an undoubted influence on those outcomes.

It should also be emphasized that in younger patients, we are working with smaller sizes of vessels than in adult patients, but most commonly, these are vessels without atherosclerotic plaques, which can impair the blood perfusion of the free flaps. Apart from a highly experienced microsurgeon, the modern anasotmotic approach with the use of a vascular coupler and relatively healthy walls of the vessels, these features mitigate achieving low rate of complications.

The most worth-mentioning modifications in the surgical technique were inclusion of the flap’s skin island as a monitor of proper bone perfusion, double microvascular venous anastomoses of the free flap to mitigate the risk of venous congestion, and modifications within the flap in cases of metaphysis involvement to avoid arthrodesis. Those modifications are described in more detail below.

### 4.1. Modifications in Cases of Metaphysis Involvement (Preventing Arthrodesis)

When the tumor infiltrates the metaphysis of the long bone, which requires resection of this part of the bone—most commonly to treat this area and preserve functionality of the joint—the endoprostheses are applied, and of course, in many cases, it is an unavoidable solution. Nevertheless, in the case of metaphyseal involvement in this study group, to avoid the implementation of artificial material, mitigating the risk of complications specific for endoprostheses and avoiding arthrodesis at the same time, we decided to harvest the fibula free flap with its head to maintain the mobility of the humeroradial joint. The head of the fibula was inserted directly into the spared annular ligament of the radius (Figure 4, marked with a green arrow).

In the other case presented in Figure 3 due to the lack of technical possibility of stabilizing the proximal part of the FFF using a titanium plate, stabilization with non-dissolvable sutures was used by drilling holes within the proximal part of the fibula, with subsequent thread insertion through the holes created and suture-fixation through the cortical layer of the remaining proximal stump of the radius. This solution also allowed for preventing arthrodesis.

### 4.2. Harvesting the FFF as Osteocutaneous Flap

All flaps were harvested, including a skin paddle, as a monitor of the transferred tissues’ viability and to avoid the necessity for performing angiographic studies to assess the blood perfusion within the “hidden” fibula.

The vast majority of studies that can be found in the literature reported FFF harvested as pure bone flaps without a skin component. These free flaps did not have a skin paddle, so they were entirely buried, without a visible viability monitor on the skin surface. What attracts attention is that in most of the studies, researchers did not perform any form of angiographic examination to assess the adequate perfusion of the free flaps in the postoperative period. This may partly explain the relatively high percentage of postoperative complications related to the bone-healing process or wound infections.

**Figure 8:**
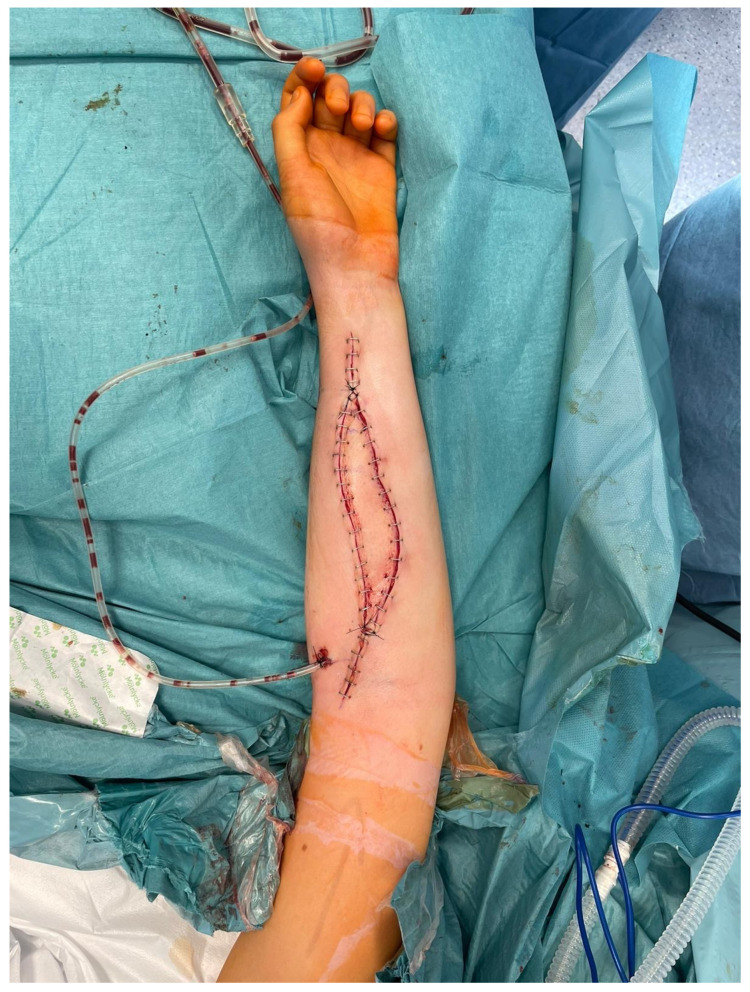
An intraoperative photograph of a skin island’s inset within the treated upper limb—a healthy skin paddle without signs of vascular congestion. Attention is drawn to the tension-free closure of the skin layers within the forearm.

It is of paramount importance, as in the case of inadequate blood supply to the transferred bone, the osseointegration of the flap with the adjacent bone stumps is considerably impaired and, in many cases, even impossible to achieve. Moreover, harvesting the skin island as a component of the FFF, apart from making tissue dissection more technically demanding and the need to identify skin perforators, does not carry any additional burden for the patient. Simultaneously, it allows for continuous monitoring of the buried bone in a safe and objective way without the need for imaging tests such as CT angiographs, which expose patients to radiation and nephrotoxicity related to the injected contrast [27,28,29]. In all cases, the donor sites were closed tension-free primarily, without the need for skin grafting (Figure 6). The skin island covering the vascularized bone graft additionally reduced the skin tension within the treated limb, which could have contributed to such a rare wound healing disorder in the analyzed group of patients (5%). Considering the above, it is difficult to find a reasonable explanation for not routinely taking into account the skin island as an external “monitor” during FFF harvest (Figure 8).

### 4.3. Double Microvascular Venous Anastomoses to Both Superficial and Deep Venous Systems

In reconstructive microsurgery, the biggest perioperative threat requiring immediate surgical intervention and frequently leading to total or partial tissue necrosis of the free flap is venous insufficiency [30]. To avoid this problem, in patients treated at our department, in order to mitigate the risk of the most common cause of flap perfusion impairment, two microsurgical veins from the FFF pedicle were anastomosed, both to the superficial and deep venous systems within the upper limb (Figure 5).

The largest meta-analysis that can be found in the literature, which included cases of microsurgical limb-sparing surgery within the lower limbs, reported that the incidence of venous insufficiency was significantly lower in the case of a flap-to-deep vein anastomosis than in the case of an anastomosis to the superficial vein [31]. Moreover, the percentage of surgical revisions after deep vein anastomosis was lower than after superficial vein anastomosis, with no statistically significant difference. In this study group, only one (5%) patient suffered from skin paddle venous insufficiency. However, this was a temporary problem that disappeared within 12 h after conservative treatment in the form of upper limb elevation (reduction of forearm swelling) as well as flap heating [32].

What should be emphasized, as excessive compression of the flap pedicle by surrounding tissues is a well-known risk factor for flap venous insufficiency, ref. [33] harvesting the above-mentioned skin paddle of the fibula free flap, which after inset of the flap significantly reduces the tension within the skin of the forearm should be considered as a protective factor for flap’s blood outflow.

## 5. Conclusions

The data provided demonstrates the efficacy of surgical excision in conjunction with prompt microsurgical reconstruction and the potential to avoid amputation following suitable patient qualifying while preserving sufficient functionality, consequently, preventing impairments in adolescents at such an early age. Every limb in this study group was spared.

The following conclusions can be made based on this study’s obtained results:

1. Patients with primary malignant bone tumors in the upper limbs can benefit from microsurgical techniques, which are highly customized, effective, and give sufficient functionality following extensive resection.

2. Despite literature reports on the applied reconstructive solution, technical modifications were reported to significantly reduce risk and even eliminate most major postoperative complications.

3. Appropriate qualification of patients for this type of procedure should be considered as an inherent part of the treatment, as primary malignant bone tumors frequently infiltrate the joint area where endoprostheses in many cases are an unavoidable solution.

The considerable heterogeneity of the bone defects resulting from resection of malignant bone tumors excludes the use of rigorous treatment algorithms in clinical practice and makes it a highly personalized procedure.

## Figures and Tables

**Table 1:** Brief characteristics and follow-up data of patients included in this study.

#	Sex	Age	Diagnosis	Bone Involved	Metaphysis Involvement	Length of the Bone Defect	Applied Reconstruction	Follow-Up (Months)	Postoperative Complications	Local Recurrence/ Distant Metastases	MSTS Scale Score (Max. 30 Points)
1	F	17 y.o.	OS	Radius	no	17 cm	FFF	45	-	-	25
2	F	13 y.o.	ES	Radius	no	14 cm	FFF	42	Wound dehiscence	-	25
3	M	17 y.o.	ES	Radius	no	18 cm	FFF	37	-	-	29
4	F	17 y.o.	OS	Ulna	no	16 cm	FFF	33	-	-	30
5	M	16 y.o.	ES	Radius	yes	19 cm	FFF	36	PIN palsy	-	19
6	F	15 y.o.	OS	Radius	no	15 cm	FFF	41	-	Lung metastases	29
7	M	13 y.o.	ES	Radius	yes	16 cm	FFF with fibular head	39	-	-	27
8	M	13 y.o.	OS	Radius	no	12 cm	FFF	34	-	-	27
9	M	14 y.o.	OS	Ulna	no	13 cm	FFF	28	-	-	30
10	F	17 y.o.	OS	Ulna	no	14 cm	FFF	27	-	-	28
11	F	13 y.o.	ES	Radius	no	15 cm	FFF	30	-	-	25
12	M	17 y.o.	OS	Ulna	no	14 cm	FFF	29	-	-	26
13	M	16 y.o.	OS	Ulna	no	16 cm	FFF	39	-	-	27
14	F	14 y.o.	ES	Radius	no	12 cm	FFF	37	-	-	24
15	M	13 y.o.	ES	Radius	no	13 cm	FFF	36	Skin paddle ischemia	-	25
16	M	15 y.o.	OS	Ulna	no	15 cm	FFF	40	-	Lung metastases	27
17	M	15 y.o.	ES	Radius	no	11 cm	FFF	36	-	-	30
18	F	15 y.o.	OS	Ulna	no	19 cm	FFF	44	-	-	25
19	F	14 y.o.	OS	Radius	no	17 cm	FFF	42	-	-	29
20	M	17 y.o.	OS	Radius	no	15 cm	FFF	27	-	-	28

OS—Osteosarcoma; ES—Ewing Sarcoma; FFF—fibula free flap; and PIN—posterior interosseus nerve.

## Data Availability

The datasets used and/or analyzed during the current study are available from the corresponding author upon reasonable request. Data are contained within the article.

## Abbreviations

3D: Three-dimensional, LSS: Limb-sparing surgery, FFF: Fibula free flap, CT: Computed Tomography, OS: Osteosarcoma, ES: Ewing Sarcoma, PIN: Posterior interosseus nerve, MSTS: Musculoskeletal Tumor Society.
